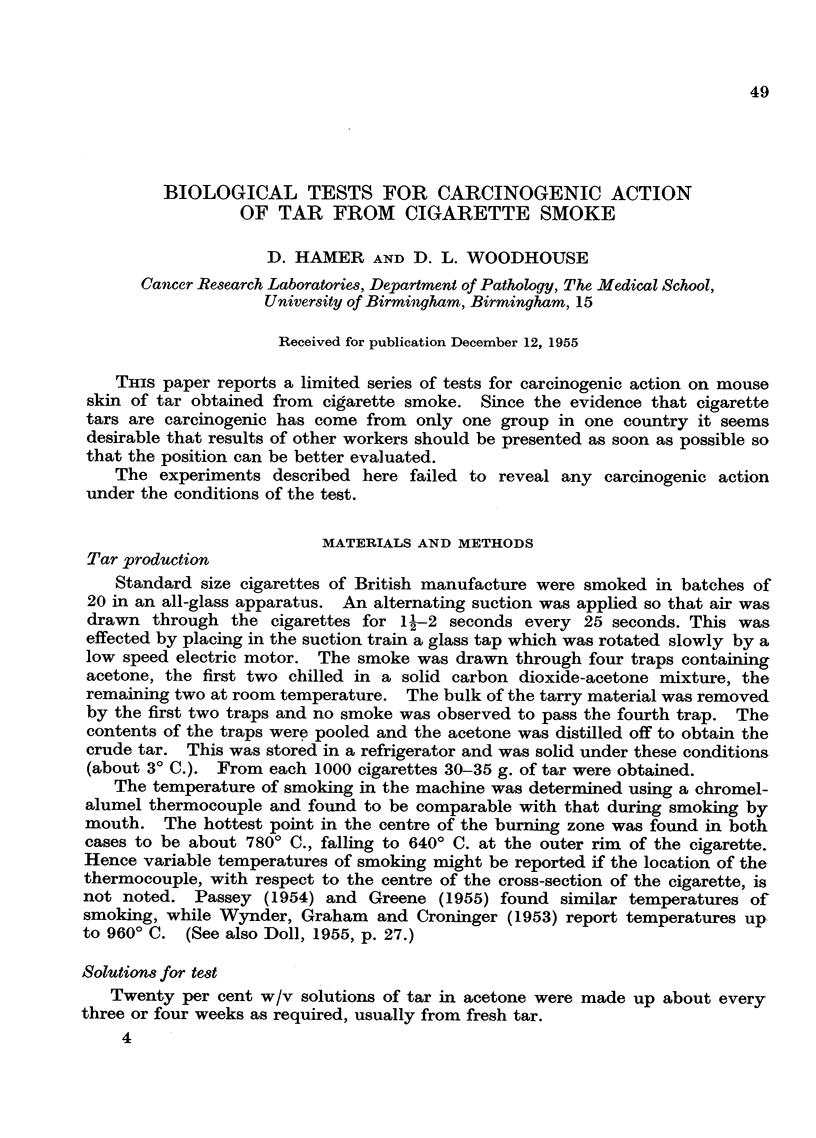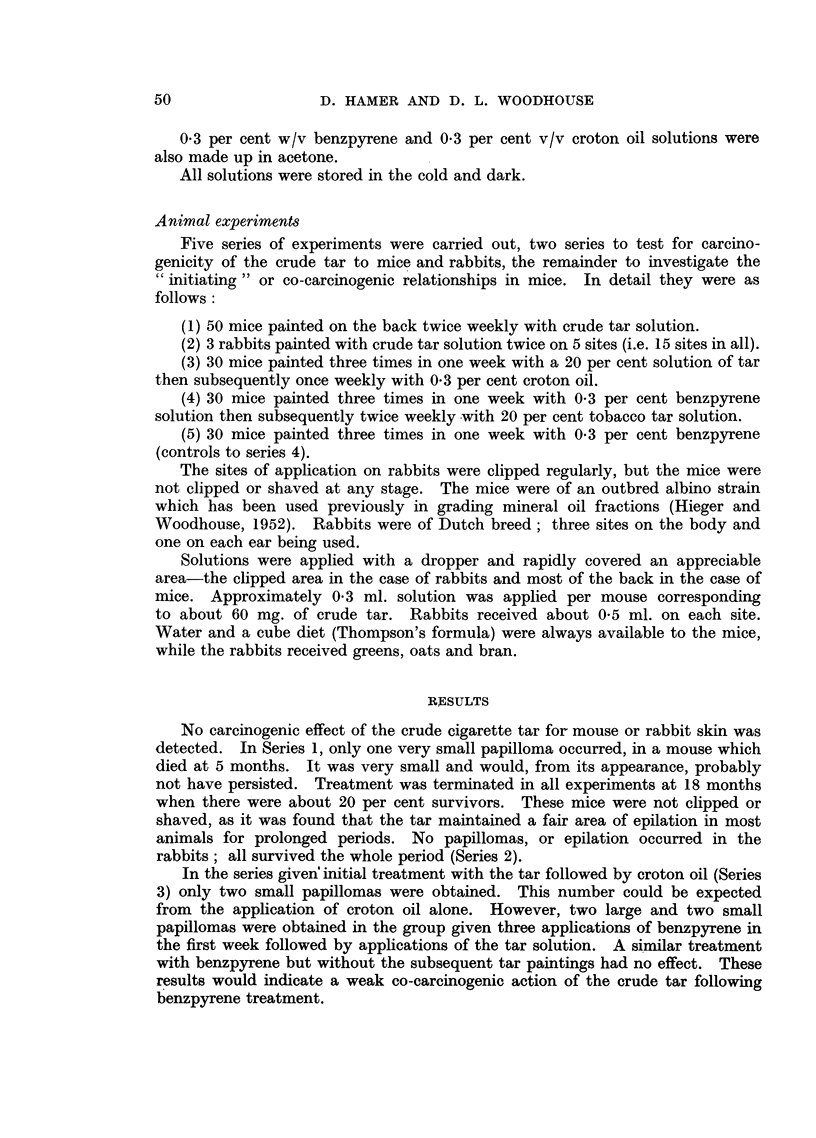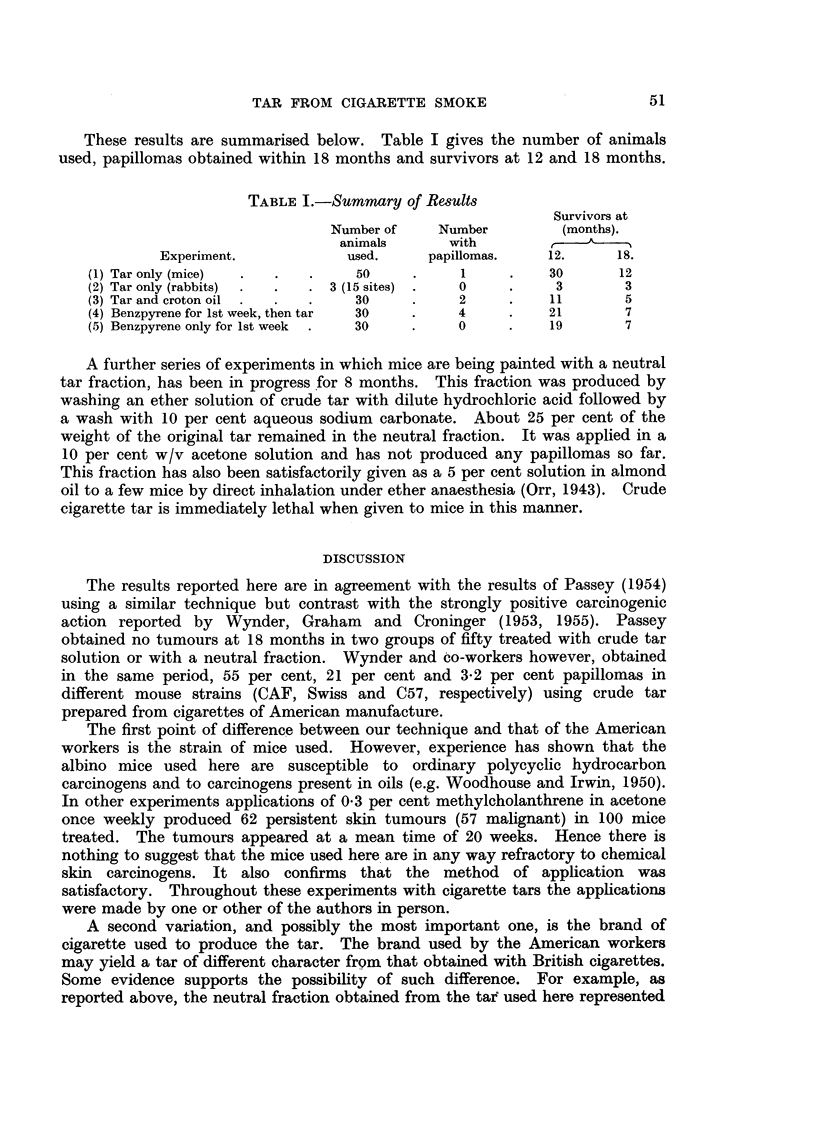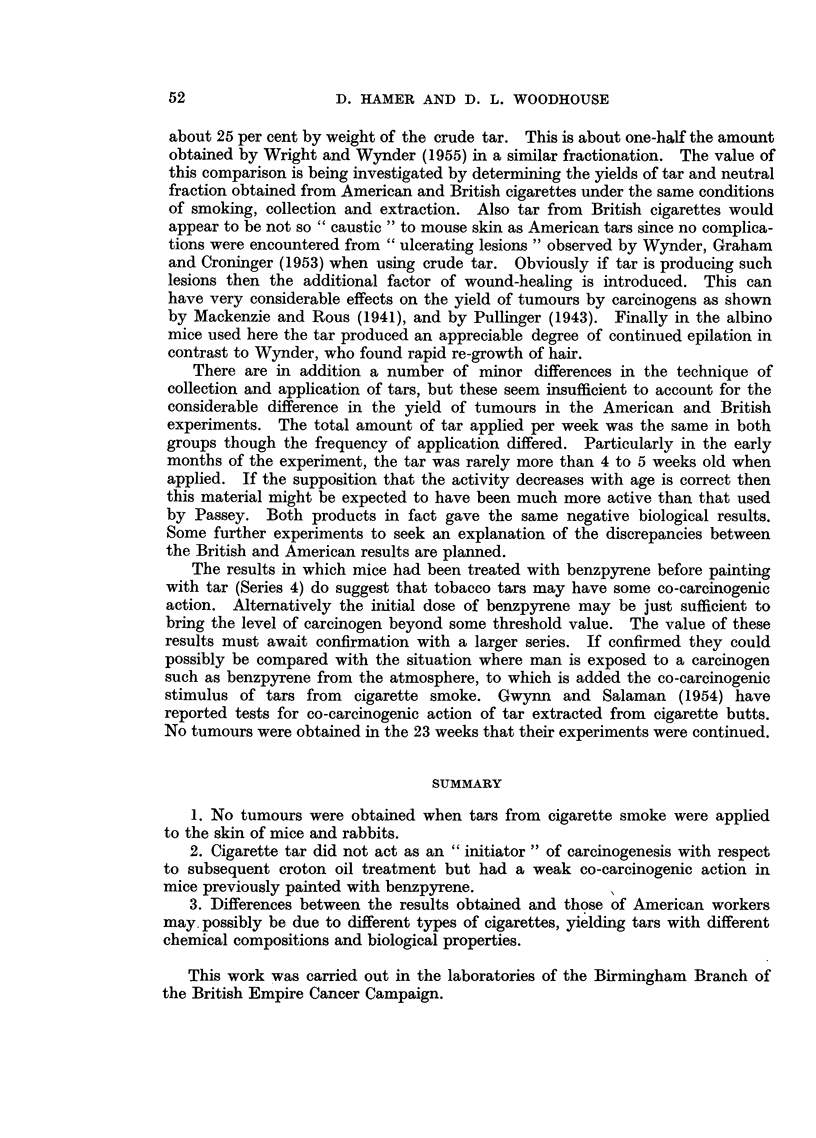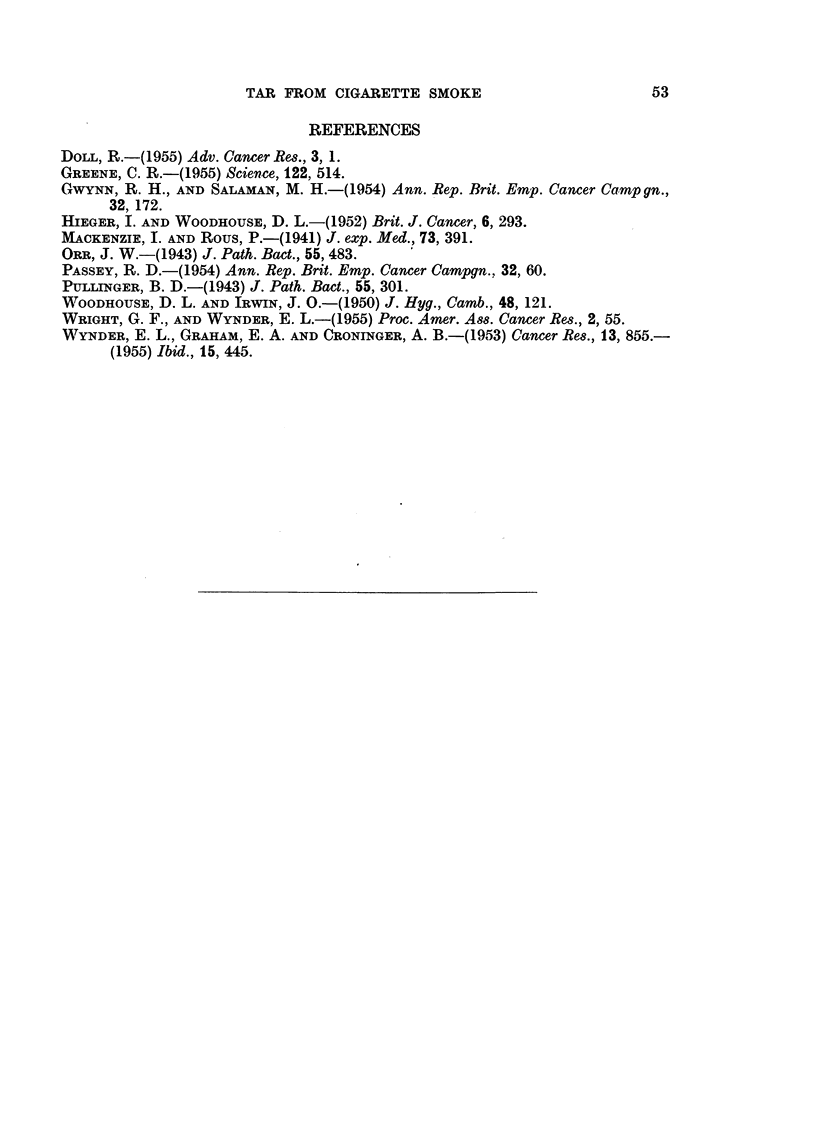# Biological Tests for Carcinogenic Action of Tar from Cigarette Smoke

**DOI:** 10.1038/bjc.1956.6

**Published:** 1956-03

**Authors:** D. Hamer, D. L. Woodhouse


### BIOLOGICAL TESTS FOR CARCINOGENIC ACTION OF TAR FROM CIGARETTE SMOKE

D. HAMER AND D. L. WOODHOUSE

*Cancer Research Laboratories, Department of Pathology, The Medical School, University of Birmingham, Birmingham,* 15

Received for publication December 12, 1955

THIS paper reports a limited series of tests for carcinogenic action on mouse skin of tar obtained from cigarette smoke. Since the evidence that cigarette tars are carcinogenic has come from only one group in one country it seems desirable that results of other workers should be presented as soon as possible so that the position can be better evaluated.

The experiments described here failed to reveal any carcinogenic action under the conditions of the test.

### MATERIALS AND METHODS

### *Tar production*

Standard size cigarettes of British manufacture were smoked in batches of 20 in an all-glass apparatus. An alternating suction was applied so that air was drawn through the cigarettes for 1½–2 seconds every 25 seconds. This was effected by placing in the suction train a glass tap which was rotated slowly by a low speed electric motor. The smoke was drawn through four traps containing acetone, the first two chilled in a solid carbon dioxide-acetone mixture, the remaining two at room temperature. The bulk of the tarry material was removed by the first two traps and no smoke was observed to pass the fourth trap. The contents of the traps were pooled and the acetone was distilled off to obtain the crude tar. This was stored in a refrigerator and was solid under these conditions (about 3° C.). From each 1000 cigarettes 30–35 g. of tar were obtained.

The temperature of smoking in the machine was determined using a chromel-alumel thermocouple and found to be comparable with that during smoking by mouth. The hottest point in the centre of the burning zone was found in both cases to be about 780° C., falling to 640° C. at the outer rim of the cigarette. Hence variable temperatures of smoking might be reported if the location of the thermocouple, with respect to the centre of the cross-section of the cigarette, is not noted. Passey (1954) and Greene (1955) found similar temperatures of smoking, while Wynder, Graham and Croninger (1953) report temperatures up to 960° C. (See also Doll, 1955, p. 27.)

### *Solutions for test*

Twenty per cent w/v solutions of tar in acetone were made up about every three or four weeks as required, usually from fresh tar.

0·3 per cent w/v benzpyrene and 0·3 per cent v/v croton oil solutions were also made up in acetone.

All solutions were stored in the cold and dark.

*Animal experiments*

Five series of experiments were carried out, two series to test for carcinogenicity of the crude tar to mice and rabbits, the remainder to investigate the " initiating " or co-carcinogenic relationships in mice. In detail they were as follows :

(1) 50 mice painted on the back twice weekly with crude tar solution.

(2) 3 rabbits painted with crude tar solution twice on 5 sites (i.e. 15 sites in all).

(3) 30 mice painted three times in one week with a 20 per cent solution of tar then subsequently once weekly with 0·3 per cent croton oil.

(4) 30 mice painted three times in one week with 0·3 per cent benzpyrene solution then subsequently twice weekly with 20 per cent tobacco tar solution.

(5) 30 mice painted three times in one week with 0·3 per cent benzpyrene (controls to series 4).

The sites of application on rabbits were clipped regularly, but the mice were not clipped or shaved at any stage. The mice were of an outbred albino strain which has been used previously in grading mineral oil fractions (Hieger and Woodhouse, 1952). Rabbits were of Dutch breed ; three sites on the body and one on each ear being used.

Solutions were applied with a dropper and rapidly covered an appreciable area—the clipped area in the case of rabbits and most of the back in the case of mice. Approximately 0·3 ml. solution was applied per mouse corresponding to about 60 mg. of crude tar. Rabbits received about 0·5 ml. on each site. Water and a cube diet (Thompson's formula) were always available to the mice, while the rabbits received greens, oats and bran.

### RESULTS

No carcinogenic effect of the crude cigarette tar for mouse or rabbit skin was detected. In Series 1, only one very small papilloma occurred, in a mouse which died at 5 months. It was very small and would, from its appearance, probably not have persisted. Treatment was terminated in all experiments at 18 months when there were about 20 per cent survivors. These mice were not clipped or shaved, as it was found that the tar maintained a fair area of epilation in most animals for prolonged periods. No papillomas, or epilation occurred in the rabbits ; all survived the whole period (Series 2).

In the series given initial treatment with the tar followed by croton oil (Series 3) only two small papillomas were obtained. This number could be expected from the application of croton oil alone. However, two large and two small papillomas were obtained in the group given three applications of benzpyrene in the first week followed by applications of the tar solution. A similar treatment with benzpyrene but without the subsequent tar paintings had no effect. These results would indicate a weak co-carcinogenic action of the crude tar following benzpyrene treatment.

These results are summarised below. Table I gives the number of animals used, papillomas obtained within 18 months and survivors at 12 and 18 months.

TABLE I.—*Summary of Results*

| Experiment. | Number of animals used. | Number with papillomas. | Survivors at (months). 12. | 18. |
|---|---|---|---|---|
| (1) Tar only (mice) | 50 | 1 | 30 | 12 |
| (2) Tar only (rabbits) | 3 (15 sites) | 0 | 3 | 3 |
| (3) Tar and croton oil | 30 | 2 | 11 | 5 |
| (4) Benzpyrene for 1st week, then tar | 30 | 4 | 21 | 7 |
| (5) Benzpyrene only for 1st week | 30 | 0 | 19 | 7 |

A further series of experiments in which mice are being painted with a neutral tar fraction, has been in progress for 8 months. This fraction was produced by washing an ether solution of crude tar with dilute hydrochloric acid followed by a wash with 10 per cent aqueous sodium carbonate. About 25 per cent of the weight of the original tar remained in the neutral fraction. It was applied in a 10 per cent w/v acetone solution and has not produced any papillomas so far. This fraction has also been satisfactorily given as a 5 per cent solution in almond oil to a few mice by direct inhalation under ether anaesthesia (Orr, 1943). Crude cigarette tar is immediately lethal when given to mice in this manner.

### DISCUSSION

The results reported here are in agreement with the results of Passey (1954) using a similar technique but contrast with the strongly positive carcinogenic action reported by Wynder, Graham and Croninger (1953, 1955). Passey obtained no tumours at 18 months in two groups of fifty treated with crude tar solution or with a neutral fraction. Wynder and co-workers however, obtained in the same period, 55 per cent, 21 per cent and 3·2 per cent papillomas in different mouse strains (CAF, Swiss and C57, respectively) using crude tar prepared from cigarettes of American manufacture.

The first point of difference between our technique and that of the American workers is the strain of mice used. However, experience has shown that the albino mice used here are susceptible to ordinary polycyclic hydrocarbon carcinogens and to carcinogens present in oils (e.g. Woodhouse and Irwin, 1950). In other experiments applications of 0·3 per cent methylcholanthrene in acetone once weekly produced 62 persistent skin tumours (57 malignant) in 100 mice treated. The tumours appeared at a mean time of 20 weeks. Hence there is nothing to suggest that the mice used here are in any way refractory to chemical skin carcinogens. It also confirms that the method of application was satisfactory. Throughout these experiments with cigarette tars the applications were made by one or other of the authors in person.

A second variation, and possibly the most important one, is the brand of cigarette used to produce the tar. The brand used by the American workers may yield a tar of different character from that obtained with British cigarettes. Some evidence supports the possibility of such difference. For example, as reported above, the neutral fraction obtained from the tar used here represented about 25 per cent by weight of the crude tar. This is about one-half the amount obtained by Wright and Wynder (1955) in a similar fractionation. The value of this comparison is being investigated by determining the yields of tar and neutral fraction obtained from American and British cigarettes under the same conditions of smoking, collection and extraction. Also tar from British cigarettes would appear to be not so " caustic " to mouse skin as American tars since no complications were encountered from " ulcerating lesions " observed by Wynder, Graham and Croninger (1953) when using crude tar. Obviously if tar is producing such lesions then the additional factor of wound-healing is introduced. This can have very considerable effects on the yield of tumours by carcinogens as shown by Mackenzie and Rous (1941), and by Pullinger (1943). Finally in the albino mice used here the tar produced an appreciable degree of continued epilation in contrast to Wynder, who found rapid re-growth of hair.

There are in addition a number of minor differences in the technique of collection and application of tars, but these seem insufficient to account for the considerable difference in the yield of tumours in the American and British experiments. The total amount of tar applied per week was the same in both groups though the frequency of application differed. Particularly in the early months of the experiment, the tar was rarely more than 4 to 5 weeks old when applied. If the supposition that the activity decreases with age is correct then this material might be expected to have been much more active than that used by Passey. Both products in fact gave the same negative biological results. Some further experiments to seek an explanation of the discrepancies between the British and American results are planned.

The results in which mice had been treated with benzpyrene before painting with tar (Series 4) do suggest that tobacco tars may have some co-carcinogenic action. Alternatively the initial dose of benzpyrene may be just sufficient to bring the level of carcinogen beyond some threshold value. The value of these results must await confirmation with a larger series. If confirmed they could possibly be compared with the situation where man is exposed to a carcinogen such as benzpyrene from the atmosphere, to which is added the co-carcinogenic stimulus of tars from cigarette smoke. Gwynn and Salaman (1954) have reported tests for co-carcinogenic action of tar extracted from cigarette butts. No tumours were obtained in the 23 weeks that their experiments were continued.

### SUMMARY

1. No tumours were obtained when tars from cigarette smoke were applied to the skin of mice and rabbits.

2. Cigarette tar did not act as an " initiator " of carcinogenesis with respect to subsequent croton oil treatment but had a weak co-carcinogenic action in mice previously painted with benzpyrene.

3. Differences between the results obtained and those of American workers may possibly be due to different types of cigarettes, yielding tars with different chemical compositions and biological properties.

This work was carried out in the laboratories of the Birmingham Branch of the British Empire Cancer Campaign.

### REFERENCES

DOLL, R.—(1955) *Adv. Cancer Res.*, **3**, 1.
GREENE, C. R.—(1955) *Science*, **122**, 514.
GWYNN, R. H., AND SALAMAN, M. H.—(1954) *Ann. Rep. Brit. Emp. Cancer Campgn.*, **32**, 172.
HIEGER, I. AND WOODHOUSE, D. L.—(1952) *Brit. J. Cancer*, **6**, 293.
MACKENZIE, I. AND ROUS, P.—(1941) *J. exp. Med.*, **73**, 391.
ORR, J. W.—(1943) *J. Path. Bact.*, **55**, 483.
PASSEY, R. D.—(1954) *Ann. Rep. Brit. Emp. Cancer Campgn.*, **32**, 60.
PULLINGER, B. D.—(1943) *J. Path. Bact.*, **55**, 301.
WOODHOUSE, D. L. AND IRWIN, J. O.—(1950) *J. Hyg., Camb.*, **48**, 121.
WRIGHT, G. F., AND WYNDER, E. L.—(1955) *Proc. Amer. Ass. Cancer Res.*, **2**, 55.
WYNDER, E. L., GRAHAM, E. A. AND CRONINGER, A. B.—(1953) *Cancer Res.*, **13**, 855.—(1955) *Ibid.*, **15**, 445.